# Incidence, indications, and risk factors for revision tibial tubercle osteotomy: A national database study

**DOI:** 10.1002/jeo2.70339

**Published:** 2025-07-24

**Authors:** Julia S. Retzky, William ElNemer, Nathan H. Varady, Vincentius J. Suhardi, Sabrina M. Strickland

**Affiliations:** ^1^ Hospital for Special Surgery New York New York USA; ^2^ School of Medicine Johns Hopkins University Baltimore Maryland USA

**Keywords:** patellofemoral instability, risk factor, revision surgery, tibial tubercle osteotomy

## Abstract

**Purpose:**

The aims of the present study are to describe the (1) incidence, (2) indications, and (3) risk factors for revision tibial tubercle osteotomy (rTTO) in a national sample of patients in the United States.

**Methods:**

A retrospective review of the Mariner PearlDiver database was performed. Patients who underwent unilateral primary tibial tubercle osteotomy (TTO) from 2015 to 2021 with a minimum 2‐year follow‐up were included, and patients who required revision TTO were identified. Patients with insufficient information, history of total knee arthroplasty (TKA), or lower extremity fracture as indication for primary TTO excluded. Demographic variables including age, sex, Elixhauser comorbidity index (ECI), diagnosis and comorbidities were compared between the no revision (NR) and revision TTO groups. Continuous variables were compared via Student's *t*‐test, and dichotomous variables were compared via chi‐squared test. Multivariate Cox‐proportional hazard modelling was utilized to identify predictors of revision TTO.

**Results:**

4919 patients who underwent TTO were included, 105 of whom required revision TTO. The average age was 26.5 ± 11.5 years, and 3782 (77%) patients were female. The median follow‐up time was 4.3 years (interquartile range [IQR]: 3.1–5.6 years). The most common indications for primary TTO included instability (62%) and chondromalacia/pain (33%). The most common indications for rTTO were instability (38%), chondromalacia/pain (28%) and fracture (24%). The median time to rTTO was 91 days [IQR: 20–219 days]. The following variables were associated with an increased risk for revision TTO: hypothyroidism (hazard ratio [HR] 1.8 [range: 1.1–3.0], *p* = 0.028), renal disease (HR = 3.3 [1.3–8.7], *p* = 0.014) and stroke (HR = 2.8 [1.3–5.8], *p* = 0.007).

**Conclusion:**

Instability is the most common indication for rTTO, and most rTTO occur within 91 days of the primary procedure. History of hypothyroidism, renal disease and stroke are all independent risk factors for rTTO. These results highlight the importance close management of higher risk patients in the perioperative period following primary TTO. Preoperative medical optimisation of conditions such as hypothyroidism may mitigate postoperative complications following primary TTO.

**Level of Evidence:**

Level III.

**Abbreviations**
ECI: Elixhauser comorbidity index
HR: hazard ratio
IQR: interquartile range
rTTO: revision tibial tubercle osteotomy
TKA: total knee arthroplasty
TTO: tibial tubercle osteotomy

## INTRODUCTION

The tibial tubercle osteotomy (TTO) is an adaptable procedure used to treat a number of pathologies affecting the patellofemoral joint [22]. For example, in the setting of patellar instability, medializing TTOs can improve patellar tracking in patients with a laterally positioned tibial tubercle [4, 5], while distalizing TTOs can improve patellar tracking in patients with patella alta [11, 14, 16, 22]. In patients with patellofemoral pain and/or chondromalacia, anteriorizing TTOs may be used to unload the joint and/or protect concurrent cartilage repair procedures [3, 9, 10, 13, 22].

Regardless of indication, several recent systematic reviews have found TTOs to be relatively safe procedures with low complication rates. For example, in a review of 787 TTOs performed primarily for coronal plane malalignment, Payne et al. [17] found a 4.6% complication rate, with infection (1%), fracture (1%) and nonunion (0.8%) being most common. Similarly, in a review of 440 knees undergoing TTO for axial plane malignment (i.e., patella alta), Knapik et al. [11] found a 7.6% complication rate, with delayed union (1.2%), thromboembolic events (0.7%) and infection (0.7%) most common.

To date, however, there is a paucity of data on the incidence, indications and risk factors for revision TTO (rTTO). This information is critical for patient counselling and could potentially inform altered postoperative rehabilitation protocols. Therefore, the purpose of this study was to describe the (1) incidence, (2) indications and (3) risk factors for rTTO in a national sample of patients in the United States.

## METHODS

**Figure 1:**
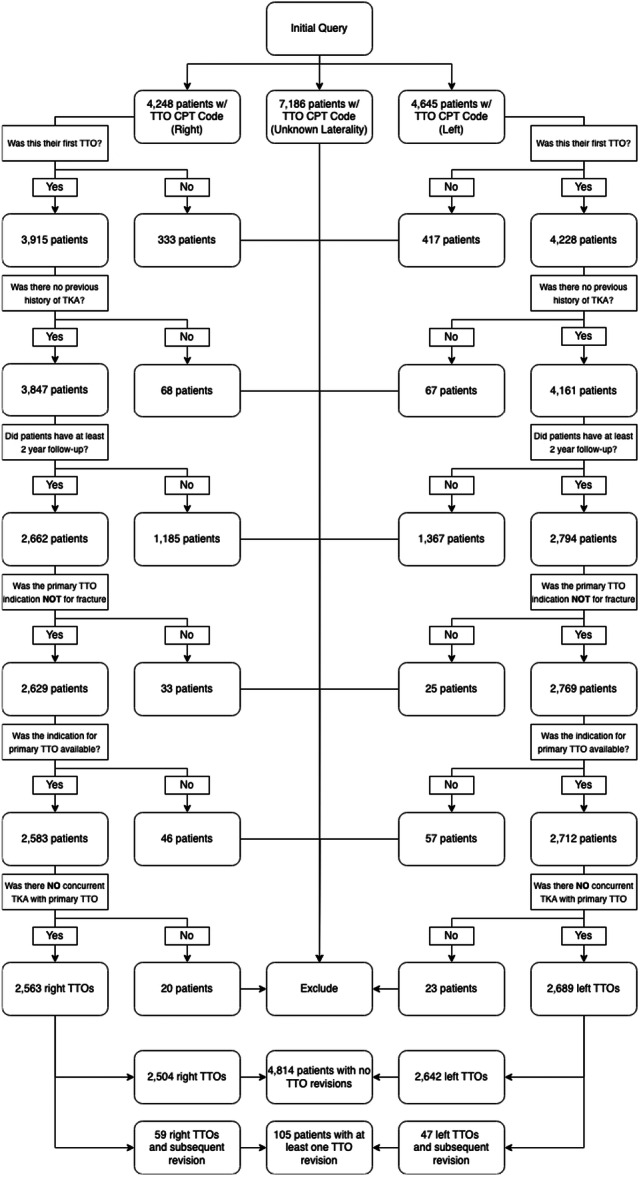
Flowchart demonstrating inclusion and exclusion criteria used in the study. CPT, current procedure terminology; TKA, total knee arthroplasty; TTO, tibial tubercle osteotomy.

A retrospective review of the Mariner database was performed through the PearlDiver software (PearlDiver Inc.). This database contains over 150 million de‐identified patient records from 2015 to 2021 with information on demographics, comorbidities and procedures over a period of follow‐up. The Current Procedure Terminology (CPT) code 27418 was used to identify patients who underwent a unilateral primary TTO. Within this cohort, patients who underwent a revision to their primary TTO (same knee) were then identified via CPT codes 27418 and 27540. Patients were eligible for the study if they had been enroled in their insurance plan for at least one year before their knee pain diagnosis and continued their enrolment for at least 2 years afterward, ensuring that data loss from early withdrawal was minimised. Patients who underwent primary TTO for fracture and patients who underwent primary TTO at the time of total knee arthroplasty (TKA) were excluded. Finally, patients who did not have information on concurrent procedures on the day as primary TTO were excluded (Figure 1). The present study was considered Institutional Review Board exempt, as patient information was de‐identified.

The indications for each patient's primary or rTTO was gathered via International Classification of Disease 10 (ICD‐10) codes. Information on concurrent procedures such as medial patellofemoral ligament reconstruction (MPFLR) and cartilaginous procedures were identified. Cartilaginous procedures included: osteochondral allograft transplantation, osteochondral autograft transplantation, cartilage debridement and matrix‐induced autologous chondrocyte implantation. Age, sex and Elixhauser comorbidity index (ECI) were collected. In addition, specific comorbidities such as smoking, hypertension, obesity, history of congestive heart failure, hypothyroidism, renal disease, sleep apnoea and stroke were also individually described.

Patients were then grouped into two cohorts: (1) patients who had a primary TTO and did not require revision (no revision [NR] group), and (2) patients who had a primary TTO and required a subsequent revision on the same knee (rTTO group). Patient characteristics were compared between these two cohorts.

Continuous variables were compared via Student's *t*‐test, and dichotomous variables were compared via chi‐squared test. A multivariate Cox‐proportion model was utilised to identify patient characteristics as independent predictors for TTO revisions. Significant variables from the univariate analyses were used as covariates in this model. Significance was set at *p* < 0.05. All statistical analyses were performed using R (version 4.3.1, R Foundation for Statistical Computing).

## RESULTS

### Demographics

In total, 4919 patients who underwent TTO were included, with a median follow‐up of 4.3 years (interquartile range [IQR]: 3.1–5.6 years). In this cohort, 3782 (77%) patients were female, and the average age was 26.5 ± 11.5 years. The most common comorbidities were obesity (1175 patients, 24%), smoking (762 patients, 15%) and hypertension (706 patients, 14%). The average ECI was 2.3 ± 2.4 (Table 1). The most common indications for primary TTO were instability (3055 patients, 62%) and chondromalacia/pain (1621 patients, 33%, Table 2). Forty‐six per cent of patients did not have any concomitant procedures at the time of primary TTO, whereas 44% of patients had a concomitant MPFLR at the time of primary TTO (Table 3).

**Table 1:** Demographics of the entire cohort and no revision versus revision subgroups.

	Full cohort (*n* = 4919)	NR (*n* = 4814)	rTTO (*n* = 105)	*p*
Age	26.5 ± 11.5	26.5 ± 11.4	25.8 ± 12.2	0.555
Sex				0.322
Male	1137 (23)	1108 (23)	29 (28)	
Female	3782 (77)	3706 (77)	76 (72)	
Follow‐up (years)	4.3 [3.1, 5.6]	4.2 [3.1, 5.5]	4.8 [3.3, 6.0]	0.304
Comorbidities				
Smoking	762 (15)	743 (15)	19 (18)	0.542
Hypertension	706 (14)	689 (14)	17 (16)	0.688
Obesity	1175 (24)	1147 (24)	28 (27)	0.576
Congestive heart failure	31 (1)	28 (1)	3 (3)	**0.022**
Hypothyroidism	497 (10)	478 (10)	19 (18)	**0.010**
Renal disease	50 (1)	46 (1)	4 (4)	**0.017**
Sleep apnoea	403 (8)	388 (8)	15 (14)	**0.034**
Stroke	120 (2)	112 (2)	8 (8)	**0.002**
ECI (mean)	2.3 ± 2.4	2.3 ± 2.4	2.7 ± 2.8	0.682
0	1139 (23)	1128 (23)	11 (10)	**0.003**
1	1133 (23)	1106 (23)	27 (26)	0.588
2	910 (18)	886 (18)	24 (23)	0.301
3	635 (13)	620 (13)	15 (14)	0.781
4	414 (8)	403 (8)	11 (10)	0.555
5	256 (5)	249 (5)	7 (7)	0.646
≥6	432 (9)	422 (9)	10 (10)	0.923

*Note*: Data are reported as *N* (%), mean ± standard deviation, or median [IQR]. Bold indicates statistical significance at *p* < 0.05.

Abbreviations: ECI, Elixhauser comorbidity index; IQR, interquartile range; NR, no revision; rTTO, revision tibial tubercle osteotomy.

**Table 2:** Indications for primary TTO for the entire cohort and no revision versus revision subgroups.

Primary TTO indications	Full cohort (*n* = 4919)	NR (*n* = 4814)	rTTO (*n* = 105)	*p*
Instability	3055 (62)	2991 (62)	64 (61)	0.885
Chondromalacia/pain	1621 (33)	1583 (33)	38 (36)	0.543
Other	243 (5)	240 (5)	3 (3)	0.443

*Note*: Data are reported as *N* (%), mean ± standard deviation, or median (IQR).

Abbreviations: NR, no revision; rTTO, revision tibial tubercle osteotomy; TTO, tibial tubercle osteotomy.

**Table 3:** Concurrent procedures during primary TTO.

Concurrent procedure at time of primary TTO	Frequency (%)
TTO only	2280 (46)
TTO + MPFLR	2165 (44)
TTO + cartilaginous	296 (6)
TTO + MPFLR + cartilaginous	178 (4)

*Note*: Cartilaginous procedures included: osteochondral allograft transplantation, osteochondral autograft transplantation, cartilage debridement and matrix‐induced autologous chondrocyte implantation.

Abbreviations: MPFLR, medial patellofemoral ligament reconstruction; TTO, tibial tubercle osteotomy.

### Analysis of cohorts

Of the 4919 patients included in the study, 105 patients (2.1%) underwent rTTO. On univariate analysis, there were no significant differences in age, sex, follow‐up, or indication for primary TTO between the NR and rTTO groups (*p* > 0.05 for all). However, the rTTO group had a high proportion of patients with hypothyroidism (18% vs. 11%, *p* = 0.015), renal disease (5% vs. 1%, *p* = 0.001), sleep apnea (16% vs. 9%, *p* = 0.018), history of stroke (8% vs. 3%, *p* = 0.018) and history of congestive heart failure (3% vs. 1%, *p* = 0.022) compared to the NR group. The rTTO group also had a lower proportion of patients with an ECI of 0 compared to the NR group (10% vs. 23%, *p* = 0.001, Table 1). The most common indications for rTTO were instability (38%), chondromalacia/pain (28%), fracture (24%) and nonunion (6%, Table 4).

**Table 4:** Indications for revision TTO at latest follow‐up and within 90 days of primary TTO.

Indications for rTTO	Latest follow‐up (*n* = 105)	90‐days postprimary TTO (*n *= 53)
Instability	40 (38)	20 (38; 50)
Chondromalacia/pain	29 (28)	7 (13; 24)
Fracture	24 (23)	20 (38; 83)
Nonunion	6 (6)	3 (6; 50)
Other	6 (6)	3 (6; 50)

*Note*: In the ‘latest follow‐up’ column, data are presented as *N* (% of total revision cohort). Data in the 90‐day postprimary TTO column are presented as *N* (% of total revision cohort within 90 days; % of patients with a specific indication revised within 90 days).

Abbreviations: rTTO, revision tibial tubercle osteotomy; TTO, tibial tubercle osteotomy.

Fifty per cent of patients underwent rTTO within 90 days of primary TTO. Of the 24 patients who underwent rTTO for fracture at any time point, 20 of the 24 patients underwent rTTO for fracture within 90 days of the primary TTO. However, of the 29 patients that underwent revision for chondromalacia/pain, only seven (24%) did so within 90 days of the primary TTO (Table 4). In addition, patients who underwent rTTO for fracture were three times more likely to have hypothyroidism compared to patients who did not undergo rTTO (7 patients [30%] vs. 478 patients [10%], *p* = 0.005). No other demographic factors were significantly different between these two groups.

### Risk factors for revision TTO

To evaluate risk factors for rTTO, we included basic demographic variables (age and sex), and comorbidities that were significant in univariate analysis (hypothyroidism, sleep apnea, renal disease and stroke) as inputs to a multivariate Cox‐proportion model. Obesity was also included in the analysis in order to correct for the known correlation for obesity with sleep apnea and hypothyroidism. Hypothyroidism (HR = 1.8 [1.1, 3.0], *p* = 0.028), renal disease (HR = 3.3 [1.3, 8.7], *p* = 0.014) and stroke (HR = 2.8 [1.3, 5.8], *p* = 0.007) were associated with increased risk of rTTO (Table 5).

**Table 5:** Multivariable cox‐proportion model for revisions.

	HR	95% CI	*p*
Age (continuous)	0.99	(0.97, 1.01)	0.192
Female sex (male ref.)	0.80	(0.52, 1.23)	0.311
Obesity (no obesity ref.)	0.91	(0.57, 1.45)	0.694
Sleep apnea (no sleep apnea ref.)	1.44	(0.79, 2.64)	0.236
Hypothyroidism (no hypothyroidism ref.)	1.80	(1.06, 3.05)	**0.028**
Stroke history (no stoke history ref.)	2.77	(1.33, 5.77)	**0.007**
Renal disease (no renal disease ref.)	3.33	(1.28, 8.72)	**0.014**

*Note*: HR > 1 indicates that younger age is more likely to have revision surgery. Bold indicates statistical significance at *p* < 0.05.

Abbreviations: HR, hazard ratio; Ref., reference.

### Survival curve

**Figure 2:**
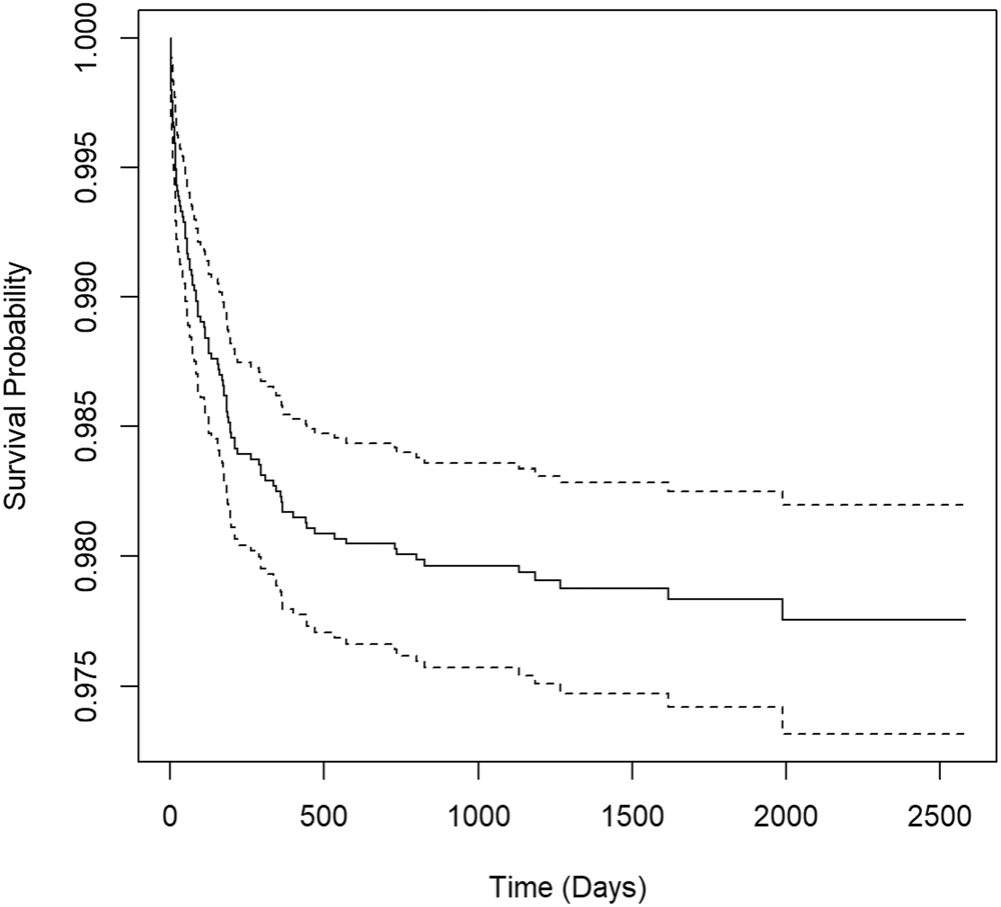
Kaplan–Meier survival curve for revision surgery. Solid lines represent probability of not requiring revision tibial tubercle osteotomy after a certain number of days. Top and bottom dashed lines represent upper and lower bounds of the 95% confidence interval of the solid curve.

Of the 105 patients who underwent rTTO, 50% of patients underwent rTTO within 91 days of the primary procedure. Seventy five percent of patients underwent revision surgery within 219 days of the primary procedure, and 90% of patients underwent revision within 469 days of the primary procedure (Figure 2).

## DISCUSSION

We found that 2.1% of patients underwent rTTO during the study period, and the most common indications for rTTO were instability, chondromalacia/pain and fracture. Hypothyroidism, renal disease and stroke were all independent risk factors for rTTO. Fifty per cent of patients underwent revision surgery within 3 months of the initial procedure. The majority of rTTO performed for fracture occurred within 90 days of the primary TTO, and a history of hypothyroidism was associated with increased TTO fracture risk.

Previous studies have identified the most common complications of primary TTO as infection [11, 17], fracture [17], nonunion/delayed union [11, 17], thromboembolic events [11] and arthrofibrosis [19]. The present study is the first to identify indications for rTTO. We found that recurrent instability was the most common indication for rTTO. Of the 3055 patients who underwent primary TTO for instability, 64 (2%) underwent rTTO for recurrent instability. This finding is in line with prior studies that have found that approximately 4% of patients who undergo primary MPFLR for patellar instability will undergo revision MPFLR for recurrent instability [20, 21]. It is possible that these patients with persistent or recurrent instability following primary TTO may have trochlear dysplasia or uncorrected patella alta at the time of the index procedure which may require increased anteromedialization and/or distalization at the time of rTTO in order to prevent recurrence.

The present study is the first to identify patient factors associated with increased risk of rTTO. Renal disease, stroke and hypothyroidism were all independent risk factors for revision surgery in the present study. There are several possible explanations for this finding. First, patients with renal disease may have hypovitaminosis D [1], which may impair healing at the osteotomy site and increase risk for nonunion and fracture. Anaemia associated with renal disease may also predispose these patients to nonunion [18] and fracture [12]. Second, stroke is closely associated with vascular disease, and inadequate vascular supply to an area of healing bone is a well‐established cause of atrophic nonunion [24]. Vascular disease may also increase fracture risk [7, 8]. Finally, recent literature has suggested that metabolic abnormalities, including endocrine derangements, are associated with increased risk for nonunion [6, 23]. Hypothyroidism is associated with hypovitaminosis D [2, 15], which may explain the predisposition to TTO fracture in this cohort. Identification of patient factors associated with increased risk of revision surgery may allow surgeons to counsel patients who may be at high risk for revision procedures appropriately preoperatively. Moreover, patients with renal disease, stroke and hypothyroidism may be targeted for preoperative medical optimisation prior to primary TTO in order to mitigate risk of future revision.

We found that more than half of patients undergo rTTO within 3 months of the primary TTO, and the majority of patients who underwent rTTO for fracture did so within 3 months of surgery. Perhaps postoperative rehabilitation protocols should be altered in order to mitigate early postoperative fracture risk in patients with hypothyroidism. Moreover, patients with hypothyroidism can be encouraged to have preoperative endocrine consultation in order to optimise thyroid hormone levels preoperatively in order to diminish risk of TTO fracture in the early postoperative period.

There are several limitations to the present study. First, this is a national database study, and the data obtained through this database is reliant on the accuracy of diagnosis and billing codes submitted. In addition, information regarding the type of TTO performed, such as anteromedializing, distalizing, or anteromedializing + distalizing, was not available in the database. However, given the relative rarity of rTTO, the use of a large database was essential to answer the study questions. The power provided by the large database is essential for identifying risk factors for rTTO, as well as providing surveillance data on the incidence and indications for rTTO in the United States.

## CONCLUSION

Recurrent instability is the most common indication for rTTO. Hypothyroidism, renal disease and stroke are all independent risk factors for rTTO. Identification of patient factors associated with increased risk for rTTO allows surgeons to counsel patients preoperatively who may be at increased risk for revision surgery. These findings also highlight the importance of preoperative medical optimisation prior to TTO.

## AUTHOR CONTRIBUTIONS

**Julia S. Retzky**: Study design; manuscript writing; manuscript editing. **William ElNemer**: Data collection; data analysis; manuscript writing. **Nathan H. Varady**: Study design; manuscript writing; manuscript editing. **Vincentius J. Suhardi**: Study design, manuscript editing. **Sabrina M. Strickland**: Study design; manuscript editing.

## CONFLICT OF INTEREST STATEMENT

Dr. Sabrina M. Strickland's disclosures below. The remaining authors declare no conflict of interest.

◦ Aesculap Biologics, LLC: Hospitality payments.
◦ Bioventus LLC: Hospitality payments.
◦ CartiHeal, Inc: Hospitality payments, research support.
◦ DePuy Synthes Sales, Inc: Hospitality payments.
◦ Dynasplint Systems, Inc: Hospitality payments.
◦ Engage Uni, LLC: Gift, stock or stock options.
◦ Flexion Therapeutics, Inc: Consulting fee, hospitality payments.
◦ Hyalex Orthopaedics, Inc: Research support.
◦ Joint Restoration Foundation, Inc: Hospitality payments, honoraria, travel and lodging, research support.
◦ Linvatec Corporation: Hospitality payments.
◦ Miach Orthopaedics, Inc: Consulting fees, hospitality payments, research support.
◦ Moximed: Consulting fees, stock or stock options.
◦ Organogenesis, Inc: Hospitality payments, research support.
◦ Pacira Therapeutics: Hospitality payments.
◦ Smith + Nephew, Inc: Consulting fees, travel and lodging, hospitality payments, compensation for services other than consulting, including serving as faculty or as a speaker at a venue other than a continuing education program, research support.
◦ Stryker: Stock or stock options.
◦ Vericel Corporation: Consulting fee, honoraria, hospitality payments, compensation for serving as faculty or as a speaker for a medical education program.

## ETHICS STATEMENT

This study was exempt from IRB approval, study reviewed by HSS IRB with confirmation of exempt status.

## Data Availability

All data are to be made publicly available.
